# Precut technique using an injection needle: A retrospective study on a new ancillary procedure for pleural biopsy

**DOI:** 10.1097/MD.0000000000029377

**Published:** 2022-08-05

**Authors:** Yasuyuki Mizumori, Katsuya Hirano, Nobuya Hirata, Ryota Hiraoka, Sayaka Takahashi, Ryota Kominami, Kohei Miyake, Masaki Takenouchi, Tomohiro Kato, Sachie Kume, Sachiko Higashino, Yasuharu Nakahara, Tetsuji Kawamura

**Affiliations:** a Department of Respiratory Medicine, National Hospital Organization Himeji Medical Center, Himeji-shi, Hyogo, Japan.

**Keywords:** cryobiopsy, injection needle, pleural biopsy, precut technique, thoracoscopy

## Abstract

The effectiveness of thoracoscopic biopsy as a diagnostic method for pleural diseases has been reported; however, obtaining a sufficient specimen size is sometimes difficult. Therefore, an ancillary technique, the precut technique using an injection needle, was devised to address this problem. This study aimed to evaluate the effectiveness and safety of the novel precut technique in patients with undiagnosed pleural effusion.

This retrospective study included 22 patients who underwent pleural biopsy using the precut technique to examine exudative pleural effusion of unknown etiology. Thoracoscopy was performed under local anesthesia. The biopsy procedure was performed as follows: a needle was inserted into the pleura around the lesion using a semiflexible thoracoscope; the needle was positioned to make an incision in the pleura while injecting 1% lidocaine with epinephrine and lifting the pleura from the fascia; 2 or 3 precut incision lines were arranged in a triangle; and the specimen was obtained from the parietal pleura using forceps or a cryoprobe. Patient data including age, number of biopsies, biopsy specimen size, pathological and final diagnosis, and postoperative complications were examined.

All patients were male with an average age of 74 years. Pleural effusion was found on the right and left sides in 16 and 6 patients, respectively. The average major axis of the biopsy specimens was 18 mm (range, 10–30 mm), which was sufficient to establish a pathological diagnosis. Only 1 patient experienced minor temporal bleeding as a complication.

The precut technique enabled the procurement of specimens sufficient in size for pleural biopsy.

## 1. Introduction

The effectiveness of thoracoscopic biopsy as a diagnostic tool for pleural diseases has been reported.^[1]^ However, the use of thoracoscopic biopsy is limited by difficulties in obtaining an appropriate specimen size using biopsy forceps. Considering this, Sasada et al^[1]^ devised a technique wherein an insulated-tip diathermic knife is used during electrocautery pleural biopsy to obtain full-thickness biopsies. Additionally, a hybrid knife with a high-pressure water jet^[2]^ and an insulated scissor-type knife^[3]^ have been reported to be effective for pleural biopsy. However, the use of electrocautery biopsy is limited by the small number of suitably skilled pulmonologists and the risk of heat-induced denaturation. Therefore, we devised the precut technique, a simple ancillary technique that uses an injection needle for pleural biopsy. Herein, we describe the procedures of the precut technique and its outcomes in a series of patients. The main aim and objectives of this study were to evaluate the effectiveness and safety of the novel and ancillary precut technique for obtaining a sufficient specimen size in patients with undiagnosed pleural effusion who underwent pleural biopsy.

## 2. Methods

### 2.1. Study population

This retrospective study was approved by the institutional review board of the local hospital. We initially considered 119 patients who underwent pleural biopsy for unilateral exudative pleural effusion of unknown etiology between April 2016 and March 2021. However, we only included patients without obvious elevated lesions because generally, localized elevated lesions of the pleura are easy to grasp with forceps, while flat lesions are not; thus, sufficiently sized specimens may not be obtained from patients with flat lesions using traditional biopsy procedures, making the use of the precut technique appropriate in such patients. Therefore, we excluded patients with obvious elevated or local lesions on chest computed tomography (CT) or thoracoscopy. Finally, 22 patients who satisfied the inclusion criteria were identified and prospectively underwent biopsy using the precut technique. Data analysis on the outcomes was performed retrospectively from patients within the study period. Informed consent for the procedure and the use of their data for publication was obtained from all patients who underwent pleural biopsy.

### 2.2. Biopsy procedures

Thoracoscopic pleural biopsy was performed under local anesthesia. The analgesic pentazocine (15 mg) was administered intravenously; no sedation was performed.

**Figure 1.**
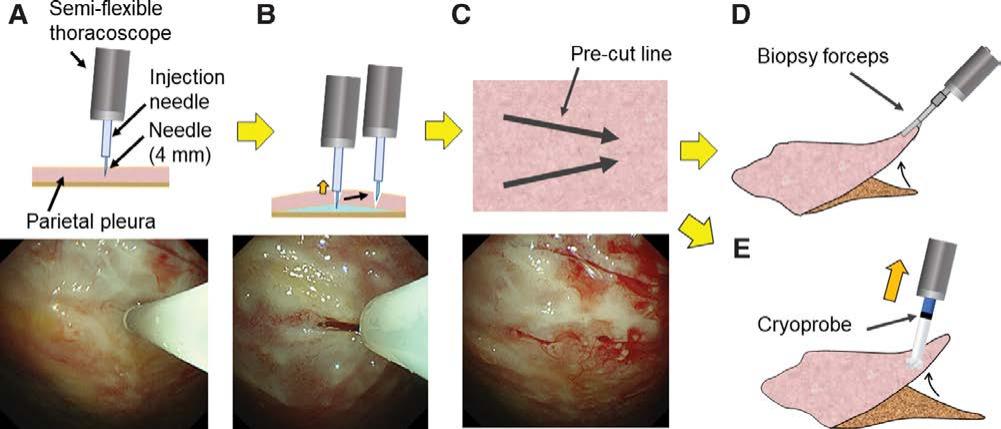
Precut procedure. (A) An injection needle (23 G, 4-mm length) is inserted into the pleura around the lesion through a semiflexible thoracoscope. (B) While injecting 1% lidocaine with epinephrine and lifting the pleura from the fascia, the needle is positioned to make an incision in the pleura. (C) Two or 3 precut incision lines are arranged in a triangle. (D) One side of the triangle is grasped by the biopsy forceps, and the specimen is peeled from the parietal pleura. (E) The specimen is obtained from the parietal pleura using a cryoprobe with a 6-s freezing time.

**Figure 2.**
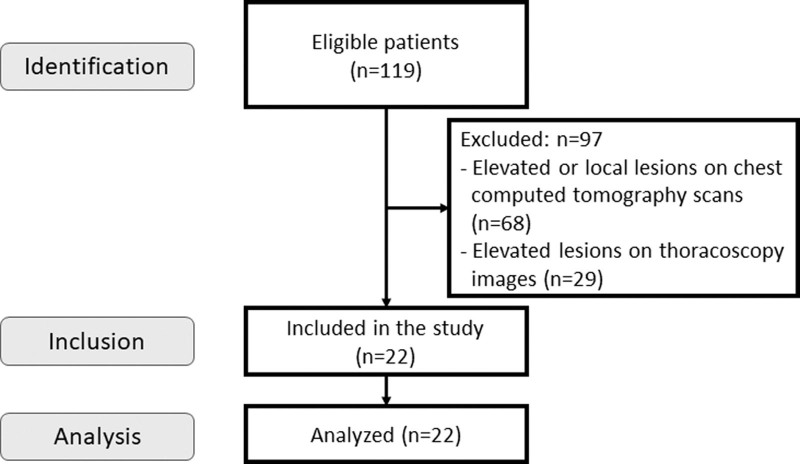
Flow diagram of patient selection.

**Figure 3.**
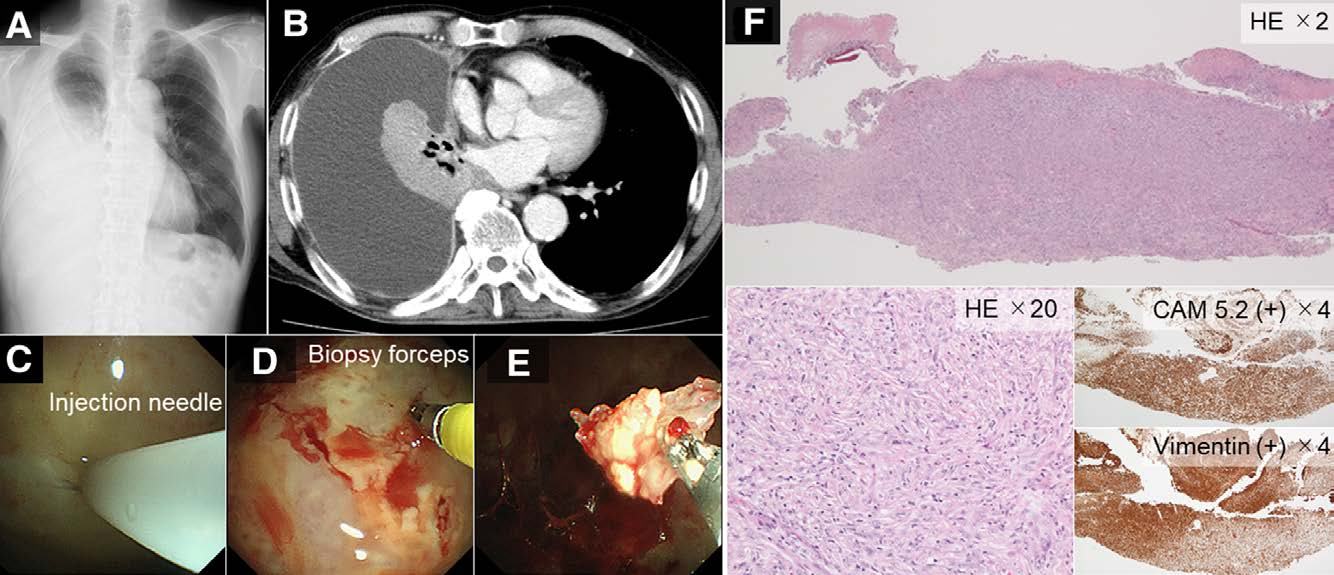
A representative case of a 76-yr-old man. (A, B) Chest radiography and computed tomography images showing right pleural effusion. (C) Using an injection needle, 3 precut incision lines are arranged in a triangle. (D) The specimen is grasped using biopsy forceps. (E) The obtained specimen. (F) The precut technique contributed to the histological diagnosis of biphasic mesothelioma. CAM = cerium ammonium molybdate staining, HE = hematoxylin and eosin staining.

The biopsy procedure was performed as follows: a needle (23 G, 4-mm length) was inserted into the pleura around the lesion guided by a semiflexible thoracoscope (LTF type 260; Olympus Ltd, Tokyo, Japan; Fig. 1A); the pleura was lifted from the fascia and the needle was positioned for incision while 1% lidocaine with epinephrine (approximately 2–3 mL) was administered (Fig. 1B); 2 or 3 triangular precut incision lines were made (Fig. 1C); and the specimen was harvested from the parietal pleura using biopsy forceps (Fig. 1D) or a cryoprobe (freezing time, 6 seconds; Fig. 1E).

The pathological diagnosis was made by microscopic evaluation of the specimen after hematoxylin and eosin staining, and immunostaining was performed if deemed necessary for diagnosis.

### 2.3. Assessments

To minimize the selection bias inherent in a retrospective study, we evaluated the effectiveness of the precut technique by excluding patients with elevated pleural lesions. For the 22 patients who underwent pleural biopsy using the precut technique, patient data regarding age, number of biopsies, biopsy specimen size, pathological and final diagnosis, and postoperative complications were retrospectively examined in August 2021 through the evaluation of electronic medical records at our hospital. This study did not include a control group, and no comparative analysis was conducted. Therefore, subgroup and sensitivity analyses were not performed. There were no missing data.

## 3. Results

All 119 patients considered for inclusion underwent pleural biopsy for unilateral exudative pleural effusion of unknown etiology. Among them, 68 patients with obvious elevated lesions or local lesions on chest CT were excluded. Of the remaining 51 patients, 29 patients who showed elevated lesions on thoracoscopy (that could not be identified by chest CT) were excluded. Finally, 22 patients who did not show any elevated lesions on thoracoscopy or CT underwent biopsy using the precut technique (Fig. 2).

The characteristics of the included patients are presented in Table 1. For pathological diagnosis, we used the precut technique for pleural biopsy followed by specimen collection using biopsy forceps in 11 patients (cf. biphasic mesothelioma [case 1 in Table 1 and Fig. 3A–F]). In the other 11 patients, cryobiopsy with the precut technique was used. The usefulness of cryoprobes in pleural biopsy has been reported.^[4,5]^ All patients were male with an average age of 74 years (range, 47–92 years). Unilateral pleural effusion was found on the right side in 16 patients and on the left side in 6 patients. Although this was a retrospective study with a small number of patients, we observed that undiagnosed pleural disease without obvious elevated lesions tended to be more common in men and on the right side. The average number of biopsy specimens obtained was 4 (range, 1–6), and the average major axis of the specimen was 18 mm (range, 10–30 mm). Immunostaining was performed in 11 patients, resulting in a pathological diagnosis of malignant mesothelioma in 5 patients, pleural metastasis of cancer in 4 patients, immunoglobulin G4-related disease in 1 patient, and fibrous pleuritis in 1 patient.

**Table 1:** Outcomes of the precut method for diagnosing pleural disease.

**Case**	**Age (yr**)	**Sex**	**Side of pleura**	**Number of biopsies**	**Maximum specimen size (mm**)	**Pathological diagnosis of the parietal pleura**	**Final clinical diagnosis**	**Complications**
1*	76	Male	Right	3	14	Mesothelioma, biphasic	Malignant mesothelioma	None
2*	72	Male	Left	4	13	Tuberculous pleuritis	Tuberculous pleuritis	None
3*	82	Male	Right	3	17	Mesothelioma, biphasic	Malignant mesothelioma	None
4*	56	Male	Right	4	21	Tuberculous pleuritis	Tuberculous pleuritis	None
5*	60	Male	Left	2	10	Chronic pleuritis	Systemic sclerosis	None
6*	76	Male	Right	4	22	IgG4-related pleuritis	IgG4-related pleuritis	None
7*	81	Male	Right	2	13	Pleuritis, no malignancy	Undiagnosed	None
8*	74	Male	Right	1	17	Metastatic adenocarcinoma	Metastatic gastric cancer	None
9*	81	Male	Right	1	15	Mesothelioma, sarcomatoid	Malignant mesothelioma	None
10*	82	Male	Right	2	26	Fibrous pleuritis	Sjögren’s syndrome	None
11*	85	Male	Left	3	20	Fibrous pleuritis	Benign asbestos pleural effusion	None
12†	70	Male	Right	6	18	Adenocarcinoma	Lung cancer	None
13†	59	Male	Left	2	30	Tuberculous pleuritis	Tuberculous pleuritis	None
14†	70	Male	Right	4	25	Eosinophilic pleuritis	Drug-induced pleuritis	Mild bleeding
15†	81	Male	Left	2	19	Fibrous pleuritis	Benign asbestos pleural effusion	None
16†	65	Male	Right	3	15	No malignancy	Pancreatic pleural effusion	None
17†	81	Male	Right	4	23	Lymphocytic infiltration	Hepatic pleural effusion	None
18†	87	Male	Right	6	15	Metastatic adenocarcinoma	Metastatic gastric cancer	None
19†	70	Male	Right	6	18	Mesothelioma, biphasic	Malignant mesothelioma	None
20†	77	Male	Right	4	20	Pleuritis, no malignancy	Undiagnosed	None
21†	77	Male	Right	5	15	Adenocarcinoma	Lung cancer	None
22†	92	Male	Left	3	13	Malignant mesothelioma	Malignant mesothelioma	None
Mean	74			4	18			None

Pathological and clinical diagnoses of all patients are shown in Table 1. Among the 22 patients, a final diagnosis was made in 20 patients (90.9%). The definitive diagnosis in all 9 patients with malignant tumor (malignant mesothelioma in 5 patients and pleural metastasis of cancer in 4 patients) was made by pleural biopsy. Of the remaining 13 patients, 11 were diagnosed with benign disease based on biopsy results and clinical course (including tuberculous pleuritis in 3 patients). The 2 patients in whom no final definitive diagnosis was made showed no progression of disease over a period of ≥1 year after thoracoscopy.

The median follow-up period was 7 months (range, 1–57 months). The outcomes of the 22 patients were as follows: 9 patients were transferred to another clinic or hospital (among them, 4 underwent treatment that resolved their condition, 3 did not show any change, and 2 were transferred to the palliative care unit), 7 patients died (6 died owing to diseases diagnosed by pleural biopsy and 1 died owing to another disease), and 6 patients continued to visit our hospital regularly.

**Figure 4.**
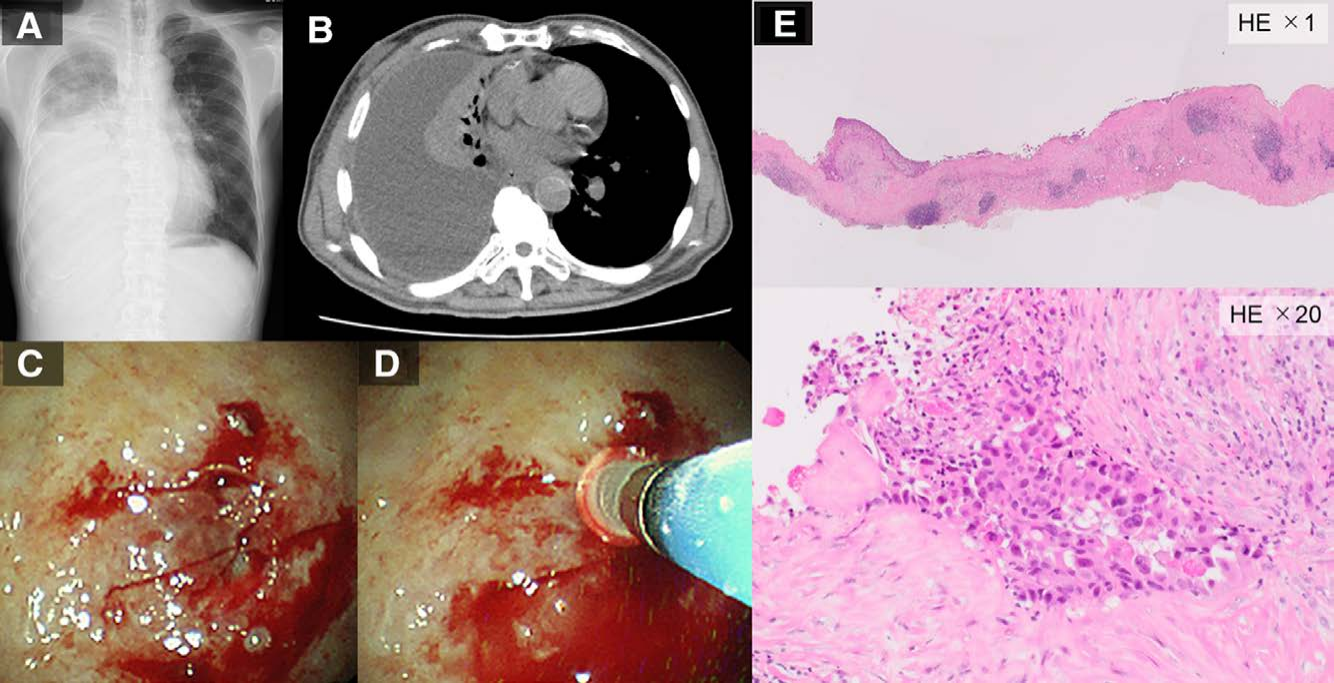
A representative case of a 70-yr-old man. (A, B) Chest radiography and computed tomography images showing right pleural effusion. (C) Image obtained after the use of the precut technique using an injection needle. (D) Freezing the specimen using a cryoprobe and peeling. (E) The precut method and cryobiopsy contributed to the histological diagnosis of adenocarcinoma. HE = hematoxylin and eosin staining.

In patient numbers 12 (Fig. 4), 15, 18, 21, and 22, the pleura was thickened and stiff; however, the specimen was harvested from the parietal pleura using the precut technique and cryoprobe.

Regarding complications, only patient number 14 experienced mild bleeding; however, hemostasis was achieved by applying pressure at the bleeding point using a cryoprobe.

## 4. Discussion

Semirigid thoracoscopy under local anesthesia is a useful diagnostic method for pleural diseases, and the diagnostic yield of thoracoscopy using biopsy forceps has been reported to be approximately 91%^[4,6]^; however, the use of thoracoscopy is limited by difficulties in obtaining an adequate specimen size, especially from thickened pleura, such as that from the mesothelioma or fibrothorax. Considering this, a new and simplified technique using an injection needle, the precut technique, was devised. In this study, the effectiveness of the precut technique as an ancillary diagnostic tool for pleural diseases was evaluated.

Although this was a retrospective study with small sample size, all 9 patients with malignant tumors were pathologically diagnosed by pleural biopsy using the precut technique. In addition, among the 13 patients without any malignancy, 4 patients (3 with tuberculous pleural inflammation and 1 with immunoglobulin G4-related disease) were pathologically diagnosed and the remaining 9 patients (of which 2 are still being followed) were diagnosed along their clinical course. Therefore, this technique can contribute to the final diagnosis of patients.

In the initial steps of this technique, 3 triangular precut incisions are made. One side of the triangle is grasped using biopsy forceps to obtain the specimen from the parietal pleura (Figs. 1A–D and 3C–E). However, in patients with thickened pleura, difficulties in generating an appropriate incision size in the pleura and performing specimen collection using biopsy forceps were evident. With the advent of the cryoprobe, a sufficient specimen size could be obtained using only 2 shallow precut incision lines, eliminating the need to cut through the entire thickness of the pleura.

Previous studies have reported the effectiveness of thoracoscopic cryobiopsy in obtaining specimens.^[4,5,7–10]^ A prospective study by Chen et al^[4]^ found a larger specimen size, better specimen quality, and higher immunostaining rate after cryobiopsy relative to after flexible forceps biopsy and closed pleural biopsy. In another prior study, 5 patients were successfully diagnosed with pleural mesothelioma using full-thickness cryobiopsy.^[5]^ These studies demonstrate the utility of the cryoprobe as a diagnostic tool for pleural biopsy. However, obtaining a sufficient specimen size using cryobiopsy can be difficult in patients with hardened and thickened pleura. Additionally, obtaining a large specimen without using the precut technique requires a considerable force that may cause pain. In the present study, no signs of pain were observed despite deferring sedation and obtaining specimens measuring an average of 18 mm. The precut technique does not necessarily involve the entire pleural tissue layer; however, pain relief is achieved because it requires less force while obtaining the desired specimen size.

Herein, the precut technique with cryobiopsy allowed us to obtain a biopsy specimen with a sufficient size in patients who presented with pleural metastasis of lung cancer (patient numbers 12 [Fig. 4] and 21), gastric cancer (patient number 18), malignant mesothelioma (patient number 22), and fibrotic pleurisy (patient number 15) who had relatively hard tissues. Conversely, in 4 patients (patient numbers 2, 4, 13, and 14) with soft pleura, the precut technique was not necessary to obtain a specimen from the pleura. However, we believe that the precut method contributed to the obtainment of larger specimens. The precut technique does not require additional time because only a sliding motion is performed during the injection of lidocaine with epinephrine.

The precut technique could be associated with an increased potential risk of vascular injury and bleeding. Risk reduction can be achieved through the following procedures: lifting the pleura while administering lidocaine and epinephrine and then slightly raising the needle to make an incision in a superficial region. In this study, 1 patient experienced mild bleeding that was managed using compression.

**Figure 5.**
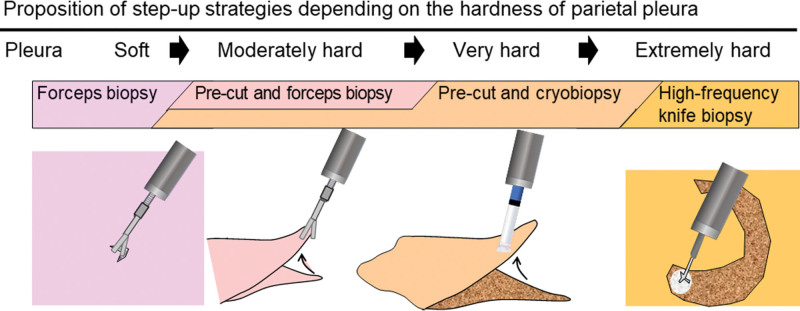
Proposition of step-up strategies depending on the hardness of the parietal pleura.

The precut technique using an injection needle may be insufficient in patients presenting with fibrotic changes that lead to an extremely hard pleura. In these patients, the use of high-frequency devices may be indicated. For routine clinical practice, the following step-up strategy should be implemented according to the pleural hardness (Fig. 5): biopsy forceps for soft pleura (easily grasped with forceps); precut technique and forceps for moderately hard pleura (barely grasped with forceps); precut technique and cryobiopsy for very hard pleura (not graspable with forceps); and high-frequency knife biopsy for extremely hard pleura (difficult to obtain a specimen even with a cryoprobe). However, for most cases, cryoprobes may be useful for obtaining pleural specimens of sufficient size in patients with pleural diseases.

Based on the results, the combination of precut technique and cryobiopsy during semirigid thoracoscopy may be a useful diagnostic procedure for pleural disease. However, a limitation of this study is its retrospective nature and small sample size. Therefore, future prospective studies with larger sample sizes are needed.

In conclusion, the precut technique is an effective, safe, and simple ancillary procedure for performing biopsy in patients with pleural diseases. Furthermore, the combination of the precut technique and cryobiopsy contributes toward diagnosing pleural diseases, even in patients with thickened and stiff pleura.

## Acknowledgments

The authors are indebted to Shinji Sasada in the Department of Respiratory Medicine, Tokyo Saiseikai Central Hospital, for his valuable advice. The authors thank Jun Kawai, Ryoko Yasumatsu, and Hiroshi Yamada in the Department of Pathology in our hospital for assisting in the pathological analysis.

## Footnotes

* Forceps biopsy,

† cryobiopsy.

## References

[1] Sasada S Kawahara K Kusunoki Y. A new electrocautery pleural biopsy technique using an insulated-tip diathermic knife during semirigid pleuroscopy. Surg Endosc. 2009;23:1901–7. 19118434 10.1007/s00464-008-0263-8

[2] Yin Y Eberhardt R Wang X-b. Semi-rigid thoracoscopic punch biopsy using a hybrid knife with a high-pressure water jet for the diagnosis of pleural effusions. Respiration. 2016;92:192–6. 27577029 10.1159/000448556

[3] Wang X-B Yin Y Miao Y. Flex-rigid pleuroscopic biopsy with the SB knife Jr is a novel technique for diagnosis of malignant or benign fibrothorax. J Thorac Dis. 2016;8:E1555–9. 28066660 10.21037/jtd.2016.11.92 PMC5179399

[4] Chen CH Cheng WC Wu BR. Feasibility and safety of pleuroscopic cryobiopsy of the pleura: a prospective study. Can Respir J. 2018;2018:6746470. 29610630 10.1155/2018/6746470 PMC5828474

[5] Nakai T Matsumoto Y Sasada S. Cryobiopsy during flex-rigid pleuroscopy: an emerging alternative biopsy method in malignant pleural mesothelioma. A comparative study of pathology. Jpn J Clin Oncol. 2019;49:559–66. 30882147 10.1093/jjco/hyz032

[6] Agarwal R Agarwal N Gupta D. Diagnostic accuracy and safety of semirigid thoracoscopy in exudative pleural effusions: a meta-analysis. Chest. 2013;144:1857–67. 23928984 10.1378/chest.13-1187

[7] Wurps H Schönfeld N Bauer TT. Intra-patient comparison of parietal pleural biopsies by rigid forceps, flexible forceps and cryoprobe obtained during medical thoracoscopy: a prospective series of 80 cases with pleural effusion. BMC Pulm Med. 2016;16:1–7. 27387441 10.1186/s12890-016-0258-5 PMC4937596

[8] Pathak V Shepherd RW Hussein E. Safety and feasibility of pleural cryobiopsy compared to forceps biopsy during semi-rigid pleuroscopy. Lung. 2017;195:371–5. 28353119 10.1007/s00408-017-9998-0

[9] Thomas R Karunarathne S Jennings B. Pleuroscopic cryoprobe biopsies of the pleura: a feasibility and safety study. Respirology. 2015;20:327–32. 25477031 10.1111/resp.12441

[10] Rozman A Camlek L Marc Malovrh M. Feasibility and safety of parietal pleural cryobiopsy during semi-rigid thoracoscopy. Clin Respir J. 2016;10:574–8. 25516173 10.1111/crj.12256

